# The distribution of herbivores between leaves matches their performance only in the absence of competitors

**DOI:** 10.1002/ece3.6547

**Published:** 2020-07-09

**Authors:** Diogo P. Godinho, Arne Janssen, Dan Li, Cristina Cruz, Sara Magalhães

**Affiliations:** ^1^ cE3c: Centre for Ecology, Evolution and Environmental Changes Faculdade de Ciências Universidade de Lisboa Lisboa Portugal; ^2^ Evolutionary and Population Biology (IBED) University of Amsterdam Amsterdam The Netherlands; ^3^ Department of Entomology Federal University of Viçosa Viçosa Brazil

**Keywords:** host‐plant quality, interspecific competition, plant defenses, spider mites, within‐plant distribution

## Abstract

Few studies have tested how plant quality and the presence of competitors interact in determining how herbivores choose between different leaves within a plant. We investigated this in two herbivorous spider mites sharing tomato plants: *Tetranychus urticae*, which generally induces plant defenses, and *Tetranychus evansi*, which suppresses them, creating asymmetrical effects on coinfesting competitors. On uninfested plants, both herbivore species preferred young leaves, coinciding with increased mite performance. On plants with heterospecifics, the mites did not prefer leaves on which they had a better performance. In particular, *T. urticae* avoided leaves infested with *T. evansi*, which is in agreement with *T. urticae* being outcompeted by *T. evansi*. In contrast, *T. evansi* did not avoid leaves with the other species, but distributed itself evenly over plants infested with heterospecifics. We hypothesize that this behavior of *T. evansi* may prevent further spread of *T. urticae* over the shared plant. Our results indicate that leaf age determines within‐plant distribution of herbivores only in absence of competitors. Moreover, they show that this distribution depends on the order of arrival of competitors and on their effects on each other, with herbivores showing differences in behavior within the plant as a possible response to the outcome of those interactions.

## Graphical abstract

Within‐plant herbivore distribution is likely to affect species interactions. Here, we show that, in absence of competitors, herbivorous spider mites settle on tomato leaves that maximize their oviposition. However, in presence of another species, they choose plant strata that minimize competition.

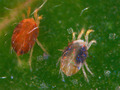

## 1 INTRODUCTION

The quality of plants as food for herbivores is highly variable, even among plants of the same species (Koricheva & Hayes, 2018; Underwood & Rausher, 2000; Wetzel, Kharouba, Robinson, Holyoak, & Karban, 2016) and among parts of the same plant (Coley, 1980; Coley, Bateman, & Kursar, 2006; Raupp & Denno, 1983). Such variation depends on traits that are intrinsic to the plant, such as the nutritional value of different tissues, or differential defenses in various tissues (Wetzel et al., 2016). Additionally, plant quality hinges on the damage caused by intraspecific and interspecific herbivores (Awmack & Leather, 2002; Karban & Baldwin, 1997).

The distribution of herbivores over different plants is expected to match differences in their performance on these plants (Jaenike, 1978; Singer, Ng, & Thomas, 1988; Thompson, 1988). Herbivore performance may be affected by different characteristics of the plant, be it physical, such as the presence of trichomes, or chemical, such as nutritional quality and inducible defenses (Awmack & Leather, 2002; Walling, 2000). For instance, the performance of herbivorous arthropods is commonly positively correlated with the amount of nitrogen in the plants (Chen, Ruberson, & Olson, 2008; Hoffland, Dicke, Van Tintelen, Dijkman, & Van Beusichem, 2000; Mattson, 1980) and negatively affected by secondary metabolites produced by plants (Bennett & Wallsgrove, 1994; Mithöfer & Boland, 2012; Walling, 2000).

Other factors can also affect host plant choice byf herbivores. For example, they may avoid plants with competitors (Pallini, Janssen, & Sabelis, 1997; Yoshimoto, 2009; Zhang, van Wieringen, Messelink, & Janssen, 2019). This may be because herbivorous arthropods alter the quality of their host plant (Awmack & Leather, 2002; Karban & Myers, 1989). Indeed, many herbivore‐induced changes in the plant are detrimental both to conspecific and heterospecific consumers, be it via the consumption of plant tissues or via the induction of plant defenses (Awmack & Leather, 2002; Kant et al., 2015; Karban & Myers, 1989; Ohgushi, 2005). To avoid the negative effects of competition, some herbivores may choose to oviposit on less crowded host plants, even if those have lower nutritional quality (Ellis, 2008; Valladares & Lawton, 1991). Alternatively, they may choose hosts where the performance of offspring is not density‐dependent such as to avoid possible costs of future over‐crowding (Wetzel & Strong, 2015). Moreover, some herbivores can also have positive effects on the performance of competitors (Awmack & Leather, 2002), for example by suppressing plant defenses (Godinho, Janssen, Dias, Cruz, & Magalhães, 2016; Matsukura, Matsumura, & Tokuda, 2012; Sarmento, Lemos, Bleeker, et al., 2011; Takemoto, Uefune, Ozawa, Arimura, & Takabayashi, 2013). These dissimilar herbivore‐induced effects on plant quality may not only result in changes of the niche of the herbivore species that modifies plant defenses, but also of that of its competitors (Hutchinson, 1959; Shimada & Fujii, 1985), and thus, affect herbivore distribution among plants.

Another factor that may affect the distribution of herbivores is the order of arrival on a plant (Peñaflor, Andrade, Sales, Silveira, & Santa‐Cecília, 2019; Stam, Dicke, & Poelman, 2018; Viswanathan, Narwani, & Thaler, 2005). The first species to colonize a plant may have a numerical advantage over its competitors, negatively affecting species that arrive later on, due to priority effects (Fukami, 2015; de Meester, Vanoverbeke, Kilsdonk, & Urban, 2016). These negative effects can be exacerbated by the induction of plant defenses. Alternatively, the presence of one species may have positive effects on the species subsequently arriving on the same host plant, facilitating its establishment (Bruno, Stachowicz, & Bertness, 2003; Callaway, 1995), which is the case of species that suppress plant defenses. In any case, the presence of a competitor may not only affect the establishment of a herbivore on a given host plant but also lead to changes in distribution at larger scales, affecting community composition (Stam et al., 2018).

Factors affecting herbivore distribution may also vary within plants (Coley, 1980; Coley et al., 2006; Meng et al., 2018; Stout, Workman, & Duffey, 1996; Travers‐Martin & Müller, 2008). For example, young, growing leaves often have higher nutritional value than old leaves, (e.g., a higher amount of N; Coley, 1980; Coley et al., 2006; Stout et al., 1996). Such differences among leaves may cause uneven performance and preference of herbivorous arthropods within a plant (Cannon & Connell, 1965; Chen et al., 2007; Cornelissen & Stiling, 2008; Opit, Margolies, & Nechols, 2003; Wiktelius, 1987). Leaves may also contain different (concentrations of) secondary metabolites, affecting the preference of herbivores, depending on whether they are negatively affected by such metabolites or can tolerate or sequester them (van der Meijden, 1996). Furthermore, some herbivores move within plants to avoid antagonists such as predators (Magalhães, Janssen, Hanna, & Sabelis, 2002; Walzer, Moder, & Schausberger, 2009) or competitors (Anderson & Agrell, 2005; Cédola, Ottaviano, Brentassi, Cingolani, & Greco, 2013; Dechert & Ulber, 2004; Gómez, Gonda‐King, Orians, & Preisser, 2014). This may benefit to herbivores, as they avoid competitors and other antagonists at low costs relative to moving to another plant. However, it may be less efficient than moving to another plant because antagonists may easily follow them within plants. Moreover, the presence of competitors in one stratum may affect plant quality in other strata (i.e., systemic plant defenses Sarmento, Lemos, Bleeker, et al., 2011; Stout et al., 1996). Even though differences in the quality of plant strata and the presence of competitors are known to affect performance of herbivores within plants, the effect of the presence of heterospecific competitors on the within‐plant distribution of herbivores has not been studied extensively (Dechert & Ulber, 2004; Gómez et al., 2014). We aimed to fill this gap by studying the performance and preference of two herbivorous spider mites coinfesting old or young leaves of the same host plant.

**FIGURE 1:**
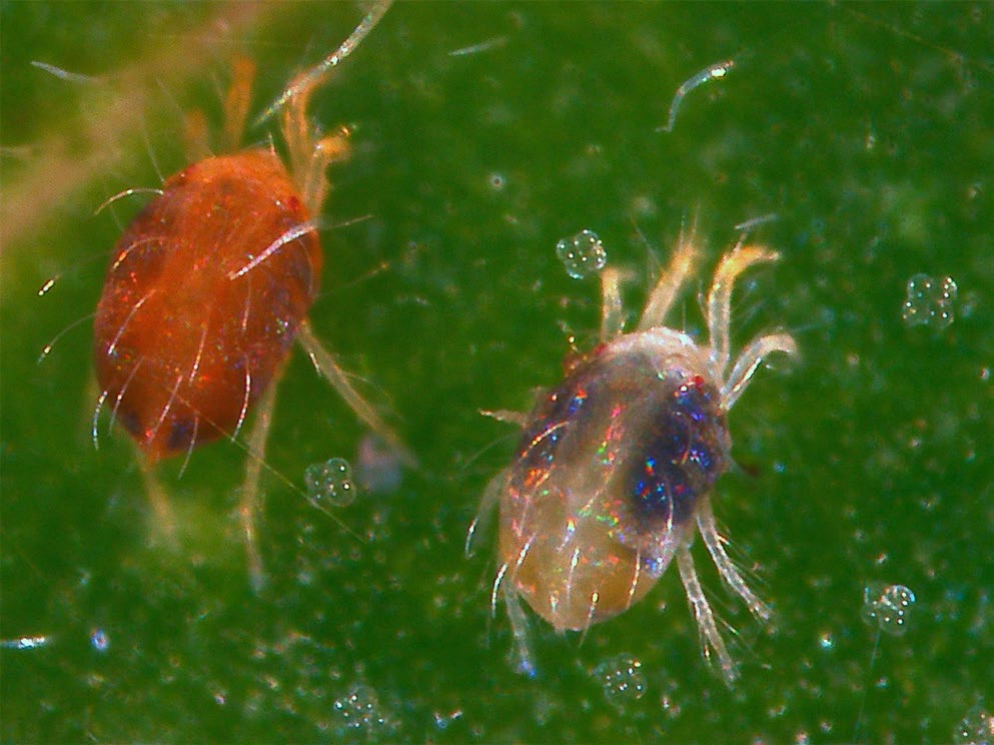
The spider mites *Tetranychus urticae* (right) and *Tetranychus evansi* (left) coinfesting a plant. Picture by Anna Beber

Spider mites (Figure 1) are herbivorous arthropod pests of many crops (Migeon, Nouguier, & Dorkeld, 2010). *Tetranychus evansi* and *T. urticae* coexist in the Mediterranean Basin, where the former is invasive (Boubou et al., 2012). *Tetranychus evansi* is a specialist of solanaceous plants and suppresses the defenses of tomato plants to levels lower than those of uninfested plants (Sarmento, Lemos, Bleeker, et al., 2011). This results in higher performance of *T. evansi*, but also of *T. urticae*, on those plants (Godinho et al., 2016; Sarmento, Lemos, Bleeker, et al., 2011; Schimmel, Ataide, Chafi, et al., 2017). In contrast, most strains of *T. urticae* induce the defenses of tomato plants, leading to lower herbivore performance on infested plants (Ament, Kant, Sabelis, Haring, & Schuurink, 2004; Kant, Ament, Sabelis, Haring, & Schuurink, 2004; Li, Williams, Loh, Gyu, & Howe, 2002). *Tetranychus urticae* and *T. evansi* thus have contrasting effects on host plant defenses. This results in oviposition rates on plants infested by the two species that are intermediate to those on plants colonized by either the inducer or the suppressor species (de Oliveira, Pallini, & Janssen, 2016, 2019; Schimmel, Ataide, & Kant, 2017). Additionally, one laboratory study shows that *T. urticae* is outcompeted by *T. evansi* on tomato plants (Sarmento, Lemos, Dias, et al., 2011). However, these previous studies did not consider how choices of herbivores for certain leaves within a plant may affect the interaction between these mite species. The distribution of mites within a plant may be affected by the presence of the interspecific competitors, by the induction or suppression of plant defenses by these competitors, but also by the quality of different plant parts for the herbivores. Addressing how interspecific competition interacts with leaf age to define the within‐plant distribution of spider mites will not only shed light on this study system, but also contribute to our general understanding of how changes in within‐plant distribution may shape herbivore communities.

## 2 MATERIALS AND METHODS

### 2.1 Mite and plant cultures

Tomato plants (*Solanum lycopersicum*, var. Moneymaker) and kidney bean plants (*Phaseolus vulgaris*, var. prelude) were sown in a 5:1 mixture of garden soil and vermiculite in a greenhouse, where they grew for 4 weeks (25°C, 16:8 LD). The plants used in the experiments had five leaves; the 2nd and 4th leaf were used for experiments and are referred to as old leaf and young leaf, respectively.

The *T. evansi* population used in these experiments was collected from tomato plants in a greenhouse in Brazil (Sarmento, Lemos, Bleeker, et al., 2011). Because *T. urticae* has a wider host range than *T. evansi* (Migeon et al., 2010), it is more likely for the former to originate from another host plant species when invading tomato plants. To account for this heterogeneity, we used a population of *T. urticae* collected on *Ricinus communis* in the Netherlands and reared on bean plants. Mites were maintained on detached leaves of tomato (*T. evansi*) or bean plants (*T. urticae*) and placed on wet cotton wool in isolated boxes under controlled conditions (25°C, 16:8 LD) in a climate room.

Cohorts of adult female spider mites were obtained by allowing groups of adult females to lay eggs on leaves of their rearing host plant for 48 hr. The adult females that emerged from those eggs were used in the experiments. All experiments were carried out in a climate‐controlled chamber under the same conditions as the rearings.

### 2.2 Effect of leaf age and infestation by heterospecific competitors on the performance of spider mites

**FIGURE A1:**
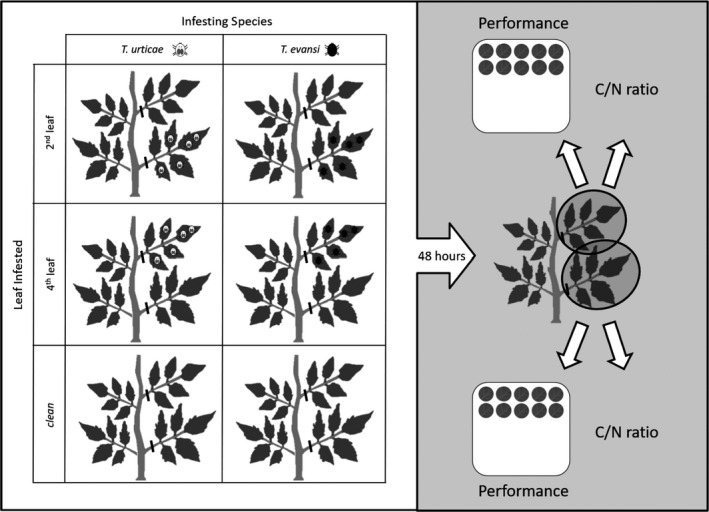
Experimental design for the measurement of the C/N ratio of each leaf and the performance of spider mites infesting those leaves. On the left panel, each square represents a different treatment. Plants were infested with either *Tetranychus urticae* or *Tetranychus evansi*, on the old leaf (2nd) or the young leaf (4th) and uninfested plants were used as control. On the right panel, the material used per plant (replicate) to collect data is represented. The performance of the species that had not previously infested each plant was determined using 10 individual discs made from each leaf, old, and young. The remaining leaf material was used to determine the C/N content

We measured the effect of heterospecifics, of leaf age, and their interaction on the performance of spider mites. Tomato plants were infested with 40 female mites of one species (*T. urticae* or *T. evansi*) either on an old leaf (2nd) or a young leaf (4th; Figure A1), while the other leaves were not infested. To prevent mites from moving to different parts of the plant, the leaves were previously isolated with lanolin paste applied to the petiole. Mites were allowed to feed and oviposit on the plants for 48 hr. Uninfested plants, similarly treated with lanolin paste, were used as control (*N* = 10 per treatment). Subsequently, females, eggs and web were removed from the leaves. Ten leaf discs of circa 4 cm^2^ were made from each leaf (old and young) of each plant (*N* = 10 per treatment; Figure A1) and placed on top of wet cotton wool in Petri dishes with the abaxial surface facing up (Figure A1). One female of the species that had not previously infested the plant was placed on each disc and allowed to oviposit for 4 days. The total number of eggs and daily survival of females were recorded. For each leaf, the average daily oviposition rate was determined by dividing the total number of eggs laid per female by the number of days on which the female was alive.

### 2.3 Effect of leaf age and infestation by heterospecific competitors on the distribution of spider mites across leaves

**FIGURE A2:**
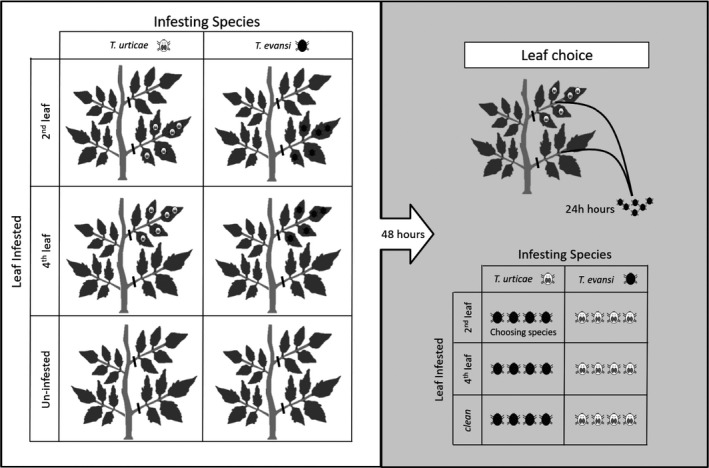
Experimental design for the choice between leaves. On the left panel, each square represents a different treatment. Plants were infested with either *Tetranychus urticae* or *Tetranychus evansi*, on the old leaf (2nd) or the young leaf (4th) and uninfested plants were used as control. On the right panel, the collection of data for the choice assay is represented. Both leaves of each plant were connected to an arena where 100 females of the species that was not infesting the plant (focal species) were released. Twenty‐four hours later, the number of females of the focal species on each leaf was registered

To assess the preference of spider mites of both species for old or young leaves, either previously infested by heterospecifics or not, plants were infested with one of the two spider mite species as in the previous experiment or left uninfested (*N* = 16 per treatment; Figure A2). After 48 hr, old and young leaves, either with spider mites and their cues or uninfested, were connected with a nylon string of equal length (35 cm) to a small Petri dish (Ø 35 mm), placed on the soil (Figure A2). Subsequently, 100 female mites of the species that was not present on the plant were released in the Petri dish and allowed to climb up the strings and choose between the leaves. Twenty‐four hours after release, the number of mites of the focal species on each leaf was recorded (Figure A2).

### 2.4 Statistics

All statistical analyses were performed with the software package R 3.0.2. Models were simplified by removing nonsignificant interactions. This was determined by comparing the full model including the nonsignificant interactions and factors, to a model excluding a given nonsignificant interaction, using the ANOVA function in R. When models were not significantly different, the model with the lowest AIC (Akaike Information Criterion) was kept (Crawley, 2007).

To assess the effect of leaf age and the presence of heterospecifics on the performance of spider mites, oviposition rates of each species, averaged per leaf, were compared using a general linear mixed‐effects model (lme). Leaf age (old or young), infestation treatment (uninfested plant, plant with old leaf infested with heterospecifics, plant with young leaf infested with heterospecifics), and their interactions were coded as fixed factors and plant was coded as a random factor. Because there was a significant interaction between leaf age and infestation treatment for *T. urticae*, differences in oviposition rate between leaves of different ages were compared for each infestation treatment using the testInteractions function (phia package, de Rosario‐Martinez, 2015).

Within uninfested plants, the preference of each species for old and young leaves was tested by comparing differences in the number of mites on each leaf, using a generalized linear model (glm) with a quasi‐Poisson error distribution to correct for overdispersion of the residuals. Tested species (*T. urticae* and *T. evansi*), leaf age, and their interaction were used as fixed factors. With this model, we also aimed to test whether the two species showed similar preference. In order to test if the preference between old and young leaves was affected by the presence of heterospecifics on one of those leaves, the distributions within plants with different infestation status were compared using a glm with a quasibinomial error distribution (to correct for the overdispersion of the residuals). The tested species (*T. urticae* or *T. evansi*), infestation treatment (as described above), and their interactions were used fixed factors. Because there was a significant interaction between the tested species and the infestation treatment, differences in the within‐plant distribution among different infestation treatments were assessed separately for each species using the testInteractions function (phia package, de Rosario‐Martinez, 2015). Additionally, the effect of the presence of heterospecifics on the proportion of mites arriving on a plant was tested using a glm with a quasibinomial error distribution (due to the overdispersion of the residuals), where the total number of mites found on the plant (old + young leaves) and the number of mites missing (i.e., the number released—the number found on the plant) were used as the response variable. The species tested (*T. urticae* or *T. evansi*), infestation treatment (coded as above), and their interactions were used as fixed factors.

## 3 RESULTS

### 3.1 Effect of leaf age and infestation by heterospecific competitors on the performance of spider mites

**FIGURE 2:**
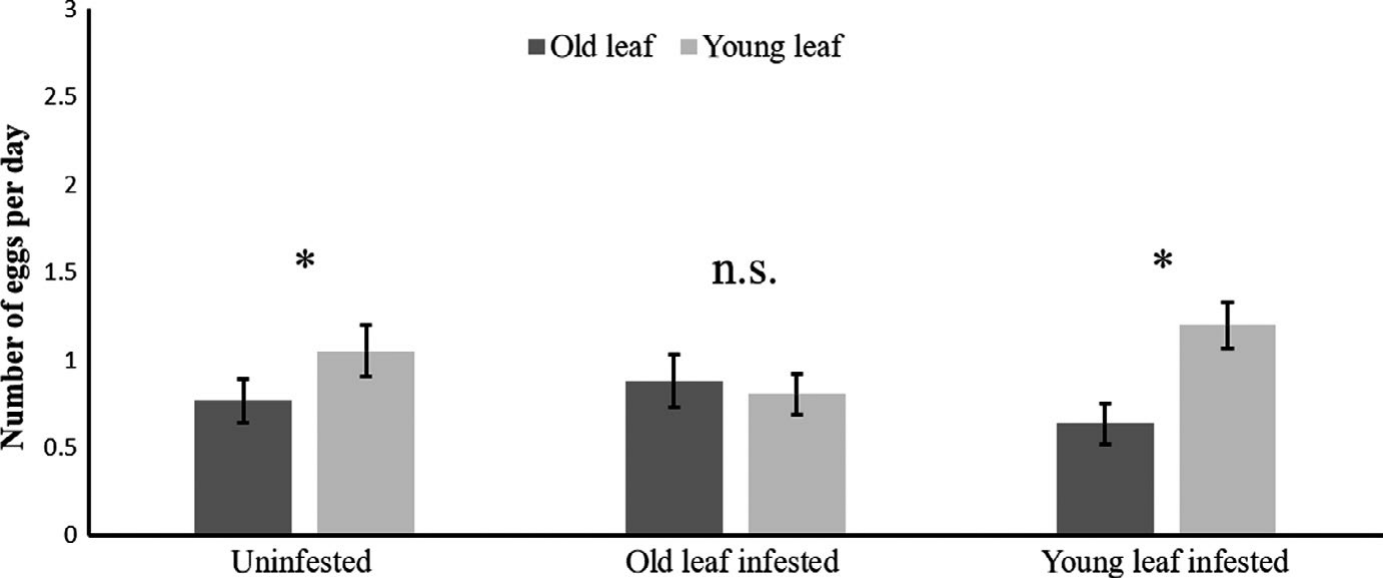
Oviposition rates of *Tetranychus urticae* on tomato plants. Average oviposition rates (eggs per female per day) of *T. urticae* (±*SE* 10 females per leaf, 10 plants) on old (dark gray) and young (light gray) leaves of tomato plants. Each set of two bars corresponds to one infestation treatment, that is, plants that were either uninfested or had previously been infested with 40 heterospecifics on the old leaf (old leaf infested) or on the young leaf (young leaf infested). Significance between leaves of different ages among treatments: *p < .05; n.s.—not significant

The oviposition rate of *T. urticae* was significantly affected by the interaction between infestation treatment and leaf age (*F*_2,10_ = 5.85, *p* = .007). The oviposition rate was 0.72‐fold higher on young leaves than on old leaves of uninfested plants (Figure 2, *χ* ^2^_1_ = 8.05, *p* = .009). On plants of which the young leaf had been infested with *T. evansi*, the same pattern was found, with the oviposition rate 0.64‐fold higher on young leaves (Figure 2, *χ* ^2^_1_ = 23.82, *p* < .001), but no such difference was found for plants of which the old leaf had been infested (Figure 2, *χ* ^2^_1_ = 0.002, *p* = .96).

**FIGURE 3:**
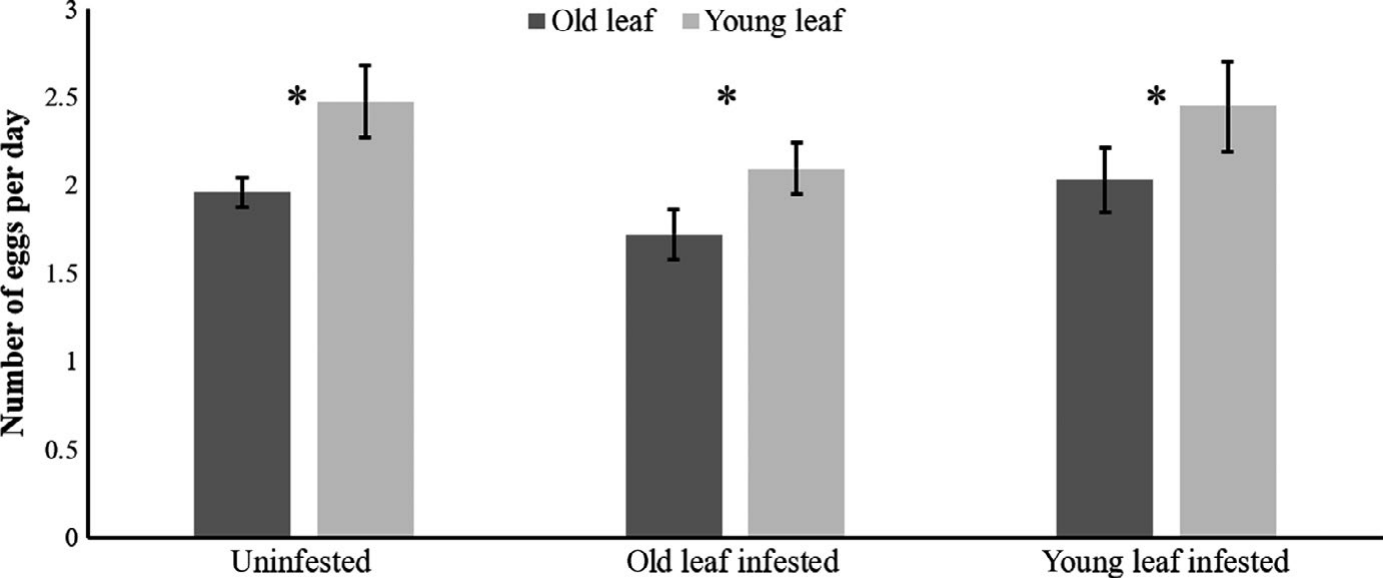
Oviposition rates of *Tetranychus evansi* on tomato plants. Average oviposition rates (eggs per female per day) of *T. evansi* (±*SE* 10 females per leaf, 10 plants) on old (dark gray) and young (light gray) leaves of tomato plants. Each set of two bars corresponds to one infestation treatment, that is, plants that were either uninfested or had previously been infested with 40 heterospecifics on the old leaf (old leaf infested) or on the young leaf (young leaf infested). Significance between leaves of different ages among treatments: *p < .05; n.s.—not significant

In contrast, the oviposition rate of *T. evansi* was not significantly affected by the interaction between infestation treatment and leaf age (*F*_2,10_ = 0.29, *p* = .74) or by previous infestation by *T. urticae* (Figure 3, *F*_2,1_0 = 0.17, *p* = .84). Overall, oviposition was significantly (1.26‐fold) higher on young than on old leaves (Figure 3, *F*_1,10_ = 33.11, *p* < .001).

### 3.2 Effect of leaf age and infestation by heterospecific competitors on the distribution of spider mites across leaves

**FIGURE 4:**
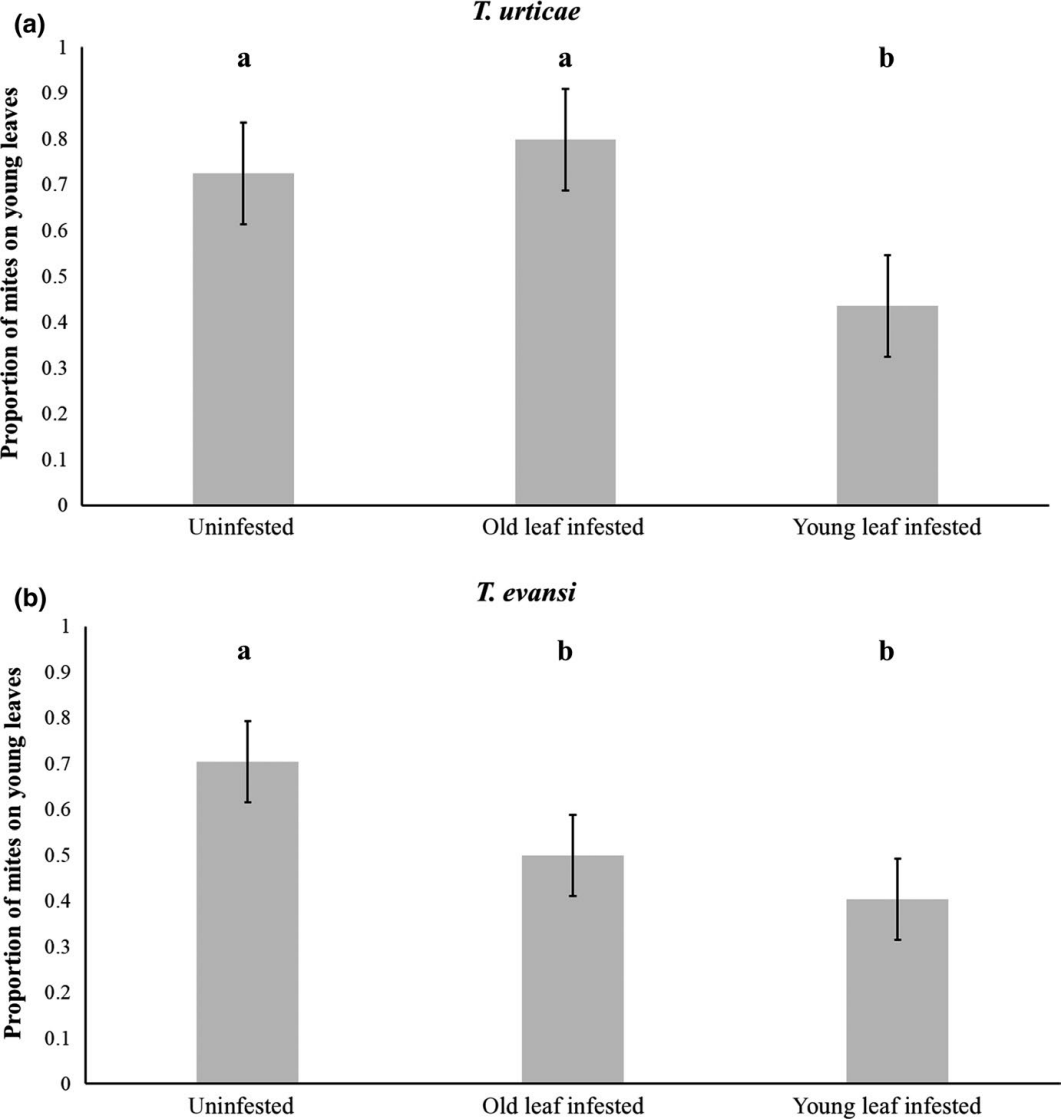
Distribution of *Tetranychus urticae* and *Tetranychus evansi* within tomato plants either uninfested or infested with heterospecifics on the old leaf or the young leaf. Shown is the proportion (±*SE*) of mites on young leaves of tomato plants (16 replicate plants per treatment; 100 mites per replicate). Plants were either uninfested or infested with 40 heterospecifics on the old leaf (old leaf infested) or on the young leaf (young leaf infested). a: Proportion of *T. urticae* on young leaves of uninfested plants or plants infested by *T. evansi*. b: Proportion of *T. evansi* on young leaves of uninfested plants or plants infested with *T. urticae*. Significant differences in preference between infestation treatments are indicated with different letters above each bar (*p* < .05)

On uninfested plants, both species showed a similar distribution across leaves (Figure 4, *F*_1,16_ = 0.01, *p* = .92); the number of *T. urticae* and *T. evansi* females was 2.51 and 2.69 fold higher on younger leaves, respectively (Figure 4, uninfested plants; *F*_1,16_ = 155.66, *p* < .001). The recovery rate, hence host acceptance, did not significantly differ between species (*F*_1,16_ = 0.33, *p* = .56) and was not affected by the presence of heterospecific competitors (*F*_2,16_ = 3.21, *p* = .28). The distribution of the mites between leaves was affected by the presence of competitors (Figure 4; *F*_2,16_ = 33.96, *p* < .001). *Tetranychus urticae* and *T. evansi* were differently affected, however, by the presence and position of the competitor on the plant (Figure 4, interaction between tested species and infestation treatment *F*_2,16_ = 16.06, *p* < .001). The distribution of the females of *T. urticae* did not differ between uninfested plants and plants of which the older leaf was infested (Figure 4a; *F*_1,16_ = 3.01, *p* = .08). In contrast, the proportion of *T. urticae* on younger leaves was 0.61‐fold lower when plants were infested with *T. evansi* than when plants were uninfested (Figure 4a; *F*_1,16_ = 44.15, *p* < .001). When younger leaves of plants were infested with *T. urticae*, the proportion of *T. evansi* females on those leaves was 0.66‐fold lower than on uninfested plants (Figure 4b; *F*_1,16_ = 59.52, *p* < .001). Additionally, when old leaves were infested with *T. urticae*, the proportion of *T. evansi* on young leaves was 0.80‐fold lower than on uninfested plants (Figure 4b; *F*_1,16_ = 32.87, *p* < .001).

## 4 DISCUSSION

This study shows that both *T. urticae and T. evansi* prefer to colonize younger rather than older leaves of uninfested tomato plants, and this was matched by higher oviposition rates on these leaves. Their preference changed when plants were infested with heterospecifics: *T. urticae* avoided leaves infested with *T. evansi*, even when the latter occupied young leaves and *T. evansi* did not avoid leaves with *T. urticae* but showed a more even within‐plant distribution than on uninfested plants. Moreover, the within‐plant distribution of spider mites did not reflect the differences in oviposition rate between leaves of different ages on plants infested with heterospecifics.

On uninfested plants, *T. urticae* had a lower oviposition rate than *T. evansi*. This was expected given that (a) this is generally the case on tomato plants (Alba et al., 2015; Sarmento, Lemos, Dias, et al., 2011; Schimmel, Ataide, Chafi, et al., 2017), and (b) our *T. urticae* population was cultured on bean, whereas *T. evansi* was reared on tomato. The higher performance and the preference of both species for young leaves, which have lower C/N ratios (Appendix 2), are in agreement with previous studies that show a preference of *T. urticae* for leaves with lower C/N ratios (Chen et al., 2007; Hoffland et al., 2000). However, other differences between leaves of different ages could explain the preference of spider mites for younger leaves. For example, differences in specific nutrients or secondary metabolites may affect the observed differences in performance and preference. In any case, our results suggest that both spider mite species preferred host plant leaves on which their oviposition rate was highest, at least in the absence of competitors. Independently of the proximate cues that trigger this behavior, the similarity in preference for younger leaves in the two mite species on uninfested plants suggests that both species will preferentially colonize the same leaves on a plant, at least in the absence of competitors. Moreover, the percentage of mites recovered on the plant was similar between species (and treatments), indicating that the ability to find a host plant does not differ substantially between these two spider‐mite species, at least not in the experimental set‐up used. Hence, the competitive interactions of the invasive species *T. evansi* with resident species such as *T. urticae* are potentially intense, as they prefer to infest the same leaves, thus having similar fundamental niches, even within plants.

On infested plants, differences in quality are not only due to differences in leaf age but are also affected by herbivory (Awmack & Leather, 2002; Karban & Myers, 1989; Walling, 2000). Here, infestation by spider mites did not affect variation in C/N ratio among different leaves (Appendix 2), confirming previous results in this system (Ximénez‐Embún, Castañera, & Ortego, 2017; Ximénez‐Embún, Ortego, & Castañera, 2016). Still, infestation by spider mites may lead to physiological changes in the plant that are not detected by this ratio (Hamilton, Zangerl, DeLucia, & Berenbaum, 2001), such as the concentration of free sugars, proteins, or amino acids (Ximénez‐Embún et al., 2016, 2017) and these, in turn, could entail differences in performance. In any case, the two spider mite species were expected to distribute themselves according to differences in their performance. However, in contrast to what was observed on uninfested plants, the distribution of mites on plants with competitors did not reflect the differences in performance on different leaves.

The population of *T. evansi* used here was shown to suppress plant defenses and this resulted in higher performance of con‐ and heterospecifics on infested leaves than on uninfested leaves (Godinho et al., 2016; Sarmento, Lemos, Bleeker, et al., 2011). Here, in contrast, the performance of *T. urticae* was not always higher on leaves infested with *T. evansi* than on uninfested leaves. Possibly, this was due to intermediate expression of plant defenses caused by the coinfestation of suppressors and inducers (de Oliveira, Pallini, & Janssen, 2016, 2019; Schimmel, Ataide, & Kant, 2017). Additionally, the effect of infestation by *T. evansi* on the performance of *T. urticae* varied with the age of the infested leaf. Indeed, infestation of old leaves by *T. evansi* led to a reduction in the differences in *T. urticae* oviposition rates on young and old leaves. By suppressing plant defenses, *T. evansi* possibly increases the quality of old leaves. As old leaves are of lower quality than young leaves, this leads to a reduction in the differences between the two types of leaves. Consequently, *T. urticae* should have less pronounced preferences for plant strata on plants where old leaves are infested by *T. evansi*. However, when *T. evansi* was present on young leaves, the differences in oviposition rates of *T. urticae* between leaves were not attenuated, because oviposition was already higher on young leaves. Based on these results, we expected *T. urticae* to still prefer young leaves when those were infested by *T. evansi*. Instead, we found that *T. urticae* avoided leaves infested with *T. evansi*. Likewise, the distribution of *T. evansi* on plants infested with heterospecifics did not match the differences in plant quality intrinsic to leaf age or differential oviposition of mites. In contrast to *T. urticae*, however, *T. evansi* distributed itself evenly between old and young leaves; hence, it did not avoid leaves with the competitor, independently of the position of the latter on the plant. Essentially, it seems that these herbivores chose leaves according to differences in their oviposition rate on uninfested plants, but not on plants infested with heterospecific competitors. Which cues trigger such differences in behavior remains to be investigated.

Possibly, spider mites did not respond to the current plant quality, but instead, to cues associated with the presence of the competitor. There is evidence that *T. urticae* is outcompeted by *T. evansi* on tomato plants (Sarmento, Lemos, Dias, et al., 2011) and that *T. evansi* interferes with the reproduction of *T. urticae* (Clemente et al., 2018). Therefore, cues associated with *T. evansi* may be indicative of the presence of a competitor that will probably impose strong fitness costs upon *T. urticae* individuals. Avoiding heterospecifics within a plant may allow *T. urticae* to increase in numbers locally, to then disperse to other host plants before being outcompeted by *T. evansi*. This may contribute to the coexistence of *T. urticae* with its invading competitor in the Mediterranean Basin (Boubou et al., 2012; Ferragut, Garzón‐Luque, & Pekas, 2013; Zélé, et al., 2018). Because *T. evansi* possibly outcompetes *T. urticae* (Sarmento, Lemos, Dias, et al., 2011), selection of *T. evansi* to avoid leaves with heterospecifics may be low, which is consistent with their behavior in our experiments. By distributing themselves evenly over different leaves in the presence of a competitor, *T. evansi* occupies more leaves, and by subsequently producing dense webbing (Sarmento, Lemos, Dias, et al., 2011), *T. evansi* can monopolize more feeding sites on a plant. In this way, it may prevent *T. urticae* from benefiting from defense suppression and from inducing defenses in these leaves (Schimmel, Ataide, & Kant, 2017). These results imply that the order of arrival is important for the outcome of the interspecific interactions between these species, as was found for other herbivorous arthropods (Erb, Robert, Hibbard, & Turlings, 2011; Huang et al., 2017; Miller‐Pierce & Preisser, 2012; Schaeffer, Wang, Thornber, Preisser, & Orians, 2018; Stam, Étien, Dicke, & Poelman, 2017), re‐enforcing the idea that priority effects may play an important role in determining the composition of herbivore communities (Stam et al., 2018).

In conclusion, we show that the distribution of herbivores within a plant is affected by the presence of heterospecifics on this plant and does not reflect their performance. Possibly, this within‐plant preference has been shaped by the asymmetries of their interspecific interactions. We speculate that these behaviors affect the outcome of those interactions and the potential for invasion. Indeed, the behavior described here for *T. evansi*, allowing monopolization of local resources, may enhance the ability to colonize novel sites (Drescher, Feldhaar, & Blüthgen, 2011; Holway, Suarez, & Case, 1998). In addition, the within‐plant avoidance behavior of *T. urticae* may contribute to the resilience of the resident herbivore community. In any case, such changes in the settling behavior may affect the distribution of species at larger spatial scales.

## CONFLICT OF INTEREST

The authors declare no conflict of interest.

## AUTHOR CONTRIBUTIONS

**Diogo P. Godinho:** Conceptualization (equal); Formal analysis (lead); Investigation (lead); Writing‐original draft (equal). **Arne Janssen:** Conceptualization (equal); Supervision (supporting); Writing‐review & editing (lead). **Dan Li:** Investigation (supporting). **Cristina Cruz:** Resources (supporting); Writing‐review & editing (supporting). **Sara Magalhães:** Conceptualization (equal); Funding acquisition (lead); Project administration (lead); Resources (lead); Supervision (lead); Writing‐original draft (equal).

## Data Availability

All data used in this work is archived in FigShare data depository, https://doi.org/10.6084/m9.figshare.12523625.

## APPENDIX 2 Effect of leaf age and spider‐mite infestation on carbon to nitrogen ratio

To assess if the C/N ratio varied among leaves of difference ages and if spider mite infestation affected this ratio, the remaining material of each leaf used in the experiment to measure the performance of spider mite was dried at 60°C until constant mass and ground. The ratio between the total carbon and nitrogen contents of old and young leaves of each plant was determined for 0.5 g of each sample, by dry combustion using an elemental analyzer (EuroVector, Italy, Rodrigues et al., 2009).

To assess the effect of leaf age and spider mite infestation on the C/N content of tomato leaves, we used a general linear mixed‐effects model (lme) with a Gaussian error distribution. C/N ratio was log transformed and used as the response variable. Leaf age (old or young), infestation treatment (uninfested plant, old leaf infested, and young leaf infested), and their interaction was used as fixed factors and replicate (plant) as a random factor. Because infestations with different species were performed separately in this experiment, using different uninfested plants as controls, this analysis was performed separately for each species.

Leaf C/N ratio was not affected by infestation with *T.urticae* (Table A1; *F*_2,10_ = 0.49, *p* = .61) independently of the age of the infested leaf (interaction between leaf age and infestation treatment: *F*_2,10_ = 0.94, *p* = .40). The same pattern was observed for *Tetranychus evansi* (Table A1; *F*_2,10_ = 2.32, *p* = .12), also independently of the age of the infested leaf (interaction between leaf age and infestation treatment: *F*_2,10_ = 0.69, *p* = .51). The C/N ratio was 1.27‐fold higher in old leaves than in young leaves, irrespective of their infestation status (Table A1; *F*_1,10_ = 87.82, *p* < .001 for infestations with *T. urticae* and *F*_1,10_ = 81.82, *p* < .001 for infestations with *T. evansi*).

**TABLE A1:** C/N content in old and young leaves of tomato plants

Species infesting	*Tetranychus urticae*			*Tetranychus evansi*		
Infestation treatment	Uninfested plant	Old leaf infested	Young leaf infested	Uninfested plant	Old leaf infested	Young leaf infested
Young leaf	5.87 ± 0.14 a	5.97 ± 0.25 a	6.33 ± 0.31 a	6.10 ± 0.22 A	6.31 ± 0.23 A	6.04 ± 0.11 A
Old leaf	7.59 ± 0.25 b	7.29 ± 0.22 b	8.45 ± 0.56 b	7.44 ± 0.20 B	7.76 ± 0.38 B	7.94 ± 0.24 B

Note

Mean C/N ratios (±*SEM*; 10 plants) in old and young leaves of tomato plants that were either uninfested or had one leaf (the old leaf or the young leaf) infested with *T. evansi* or *T. urticae*. Different letters indicate significant differences (lower case letters for plants infested with *T. urticae* and capital letters for plants infested with *T. evansi*).
